# Membrane localization of the Repeats-in-Toxin (RTX) Leukotoxin (LtxA) produced by *Aggregatibacter actinomycetemcomitans*

**DOI:** 10.1371/journal.pone.0205871

**Published:** 2018-10-18

**Authors:** Angela C. Brown, Kathleen Boesze-Battaglia, Nataliya V. Balashova, Nestor Mas Gómez, Kaye Speicher, Hsin-Yao Tang, Margaret E. Duszyk, Edward T. Lally

**Affiliations:** 1 Department of Pathology, University of Pennsylvania, School of Dental Medicine, Philadelphia, PA, United States of America; 2 Department of Biochemistry, University of Pennsylvania, School of Dental Medicine, Philadelphia, PA, United States of America; 3 Wistar Institute, Philadelphia, PA, United States of America; Medical University of South Carolina, UNITED STATES

## Abstract

The oral bacterium, *Aggregatibacter actinomycetemcomitans*, which is associated with localized aggressive periodontitis, as well as systemic infections including endocarditis, produces numerous virulence factors, including a repeats-in-toxin (RTX) protein called leukotoxin (LtxA), which kills human immune cells. The strains of *A*. *actinomycetemcomitans* most closely associated with disease have been shown to produce the most LtxA, suggesting that LtxA plays a significant role in the virulence of this organism. LtxA, like many of the RTX toxins, can be divided into four functional domains: an N-terminal hydrophobic domain, which contains a significant fraction of hydrophobic residues and has been proposed to play a role in the membrane interaction of the toxin; the central domain, which contains two lysine residues that are the sites of post-translational acylation; the repeat domain that is characteristic of the RTX toxins, and a C-terminal domain thought to be involved in secretion. In its initial interaction with the host cell, LtxA must bind to both cholesterol and an integrin receptor, lymphocyte function-associated antigen-1 (LFA-1). While both interactions are essential for toxicity, the domains of LtxA involved remain unknown. We therefore undertook a series of experiments, including tryptophan quenching and trypsin digestion, to characterize the structure of LtxA upon interaction with membranes of various lipid compositions. Our results demonstrate that LtxA adopts a U-shaped conformation in the membrane, with the N- and C-terminal domains residing outside of the membrane.

## Introduction

*Aggregatibacter actinomycetemcomitans* is a facultative anaerobic bacterium commonly found in the upper aerodigestive tract of man and certain higher primates [1]. The organism produces a 114-kDa RTX (Repeats in ToXin) toxin [2] or leukotoxin (LtxA) that expresses a specificity that is unique to human immune cells [3–5].

**Fig 1:**
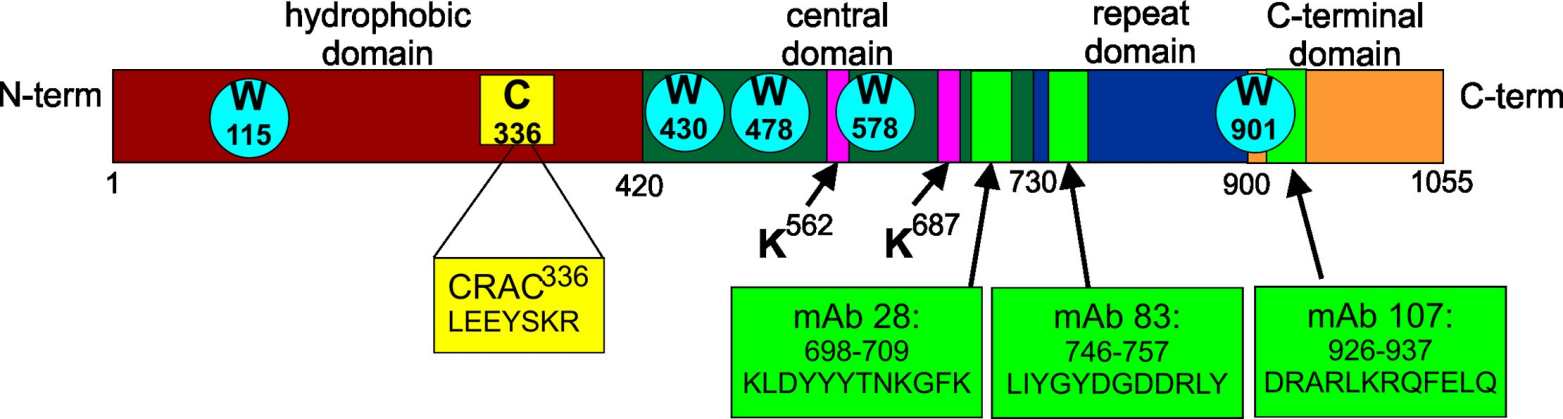
Four functional domains of LtxA. The hydrophobic domain (residues 1–420, red) contains most of the hydrophobic amino acid residues of LtxA. The central domain (residues 421–730, green) contains two internal lysine residues (K^562^ and K^687^, bars, K) that are the sites of post-translational acylation. The repeat domain (residues 731–900, blue) contains the characteristic repeated amino acid sequence of the RTX family. The C-terminal domain (residues 901–1055, orange) is hypothesized to play a role in secretion. A cholesterol binding site (CRAC^336^, yellow square, C) is located in the hydrophobic domain. Five tryptophan residues (blue circles, W) are located throughout the sequence of LtxA at residues 115, 430, 478, 577, and 901. The three monoclonal antibody epitopes are marked with green bars; mAb 28 recognizes residues 698–709, mAb 83 recognizes residues 746–757, and mAb 107 recognizes residues 926–937 [14].

The RTX toxins are a family of large, bacterial proteins with diverse biological functions that are produced by an ever increasing number of Gram-negative bacteria [2]. This family of toxins possesses a number of similarities, including sequence and structural homology, which has allowed us to divide LtxA into four functional domains (Fig 1) for our studies. The hydrophobic domain (residues 1–420) contains most of the hydrophobic amino acid residues found in LtxA [6] and because of this, this region has historically been proposed to engage the membrane in some manner [7]. The central domain (residues 421–730) contains two lysine residues, K^562^ and K^687^, that are the sites of the post-translational acylation [8]. The repeat region (residues 731–900) contains fourteen nonapeptide, glycine-rich repeated amino acid sequences, which fold into a β-roll conformation in the presence of calcium [9–12]. Finally, the C-terminal domain (residues 901–1055) has been suggested to be involved in secretion [13].

RTX toxins also share a mechanism of secretion which utilizes the type 1 secretion system (T1SS) [2], an oligomeric channel that spans the bacterial cell envelope and enables single-step translocation of RTX polypeptides from the bacterial cytosol across both the inner and the outer bacterial membrane, directly into the extracellular space without involvement of periplasmic secretion intermediates [15–17]. The ability of the toxin to be secreted via the T1SS is regulated by the calcium-binding activity of the repeat domain [18]. In the bacterial cytoplasm, the concentration of Ca^2+^ ions is typically low (<100 nM) [19] allowing for maintenance of an RTX repeat region that is flexible and amenable to T1SS-mediated secretion. Once in the extracellular space that is colonized by the oral microflora, millimolar calcium concentrations are typically encountered [20], which results in loading of the RTX repeat domain of the secreted protein by Ca^2+^ ions [21, 22], completion of tertiary folding [10, 23], and acquisition of biological activity [18].

Once secreted into the external environment, LtxA travels in a water-soluble form through this milieu before recognizing and binding to white blood cells. The recognition of its human host by LtxA is multifaceted and involves both the membrane lipids [24, 25] and a cell-surface glycoprotein, lymphocyte function-associated antigen-1 (LFA-1), a β_2_ integrin family member [26]. The cell specificity of LtxA derives from its association with LFA-1 [26, 27], which is uniquely expressed on the plasma membranes of immune cells [26]. While the interaction of LtxA with LFA-1 is essential for activity of the toxin, LtxA also requires the presence of cholesterol in the host plasma membrane, a recognition process that is mediated by the presence of a cholesterol recognition amino acid consensus (CRAC) motif [24], which is defined by the sequence -L/V–X_1-5_–Y–X_1-5_–R/K- [28, 29] and is located within the hydrophobic domain of LtxA. The interaction of LtxA with LFA-1 and the plasma membrane lipids appears to be linked, as the toxin’s binding to cholesterol initiates a signaling cascade that leads to the clustering of LtxA and LFA-1 in large cholesterol-rich rafts [8]. Additionally, model membrane experiments suggest that LtxA associates with immune cell membranes to form round, toxin-rich patches and lipid-lined cavities through a process involving membrane bending [25]. Similar interactions have been observed for a number of RTX toxins, including *Bordetella pertussis* adenylate cyclase toxin [30] and *Mannheimia haemolytica* leukotoxin [31].

LtxA epitopes on LFA-1 have been proposed to reside within β sheets 1 and 2 of the β-propeller of the α_L_ subunit of LFA-1 [32, 33] and integrin-epidermal growth factor-like domains 2, 3, and 4 of the β_2_ subunit [34] of LFA-1. In addition, we have recently discovered that LtxA binds to the cytosolic α_L_ and β_2_ domains of LFA-1 [35], suggesting that LtxA may reside partially embedded in the membrane, allowing it to interact with both extracellular and intracellular domains of both subunits of LFA-1.

It appears that the interaction of the RTX toxins with the host cell plasma membrane is shared among the various toxins; however since the molecular structure of an RTX protein has not been solved, the specific domains of the toxins that interact with the membrane have not been identified beyond our identification of the cholesterol-binding site [24]. The reasons for this issue are not clear and could reside in the intrinsic disorder that is naturally present in the nonapeptide repeats or the posttranslational modifications the protein undergoes prior to secretion. Our goal in the current study was to use a variety of biophysical techniques, including tryptophan quenching, trypsin digestion, and mass spectrometry to determine the orientation of LtxA on the host cell plasma membrane to better understand the toxin’s membrane and receptor interactions.

## Materials and methods

### LtxA purification

*A*. *actinomycetemcomitans*, strain JP2, was grown overnight in AAGM broth [36] and the secreted toxin was purified following a published protocol [37]. The bacterial culture supernatant was precipitated with ammonium sulfate (32.5%, 4°C, 2 h). The precipitate was recovered by centrifugation (10,000 r.p.m., 20 min), suspended in phosphate buffer (10 mM KH_2_PO_4_, pH 6.5), and dialyzed overnight in phosphate buffer. The supernatant was then filtered and applied to a HiTRAP SP column (GE Healthcare, Piscataway, NJ, USA). LtxA was eluted with a buffer containing 0.6 M NaCl in 10 mM KH_2_PO_4_, pH 6.5. The toxin was lyophilized and stored at -80°C. The purity of LtxA was confirmed by SDS-PAGE, the identity by Western blot, and the activity with a cytotoxicity assay [37] (S1 Fig).

### Cell culture

The T lymphocyte cell line, Jn.9, a subclone of Jurkat cells [38], were maintained in RPMI 1640 medium containing 10% FBS, 0.1 mM MEM non-essential amino acids, 1x MEM vitamin solution, 2 mM L-glutamine, and 0.5 μg/mL gentamicin at 37 °C under 5% CO_2_.

### Immunofluorescence

LtxA was labeled with DyLight^TM^ 650 (LtxA-DY650) using DyLight^TM^ Amine-Reactive dye (Pierce) according to the manufacturer’s instructions. After labeling, the excess dye was washed using a Zeba^TM^ Spin Desalting column (40k MWCO, ThermoFisher^TM^ Scientific). For LtxA and cell membrane interaction studies, 1 x 10^6^ Jn.9 cells were incubated with 20 nM LtxA-DY650 for 30 min at 37 °C in a 24-well plate. We selected the 20 nM concentration for our experiments because it is a sub-maximal dose at which the effect of LtxA on Jn.9 cells is pronounced and specific. The cells were washed with PBS, and the cell nuclei and plasma membranes were labeled for 10 min with 1 μM Hoechst 33342 and 5 μg/mL Wheat Germ Agglutinin AlexaFluor^TM^ 488 conjugate (WGA-AF488, Invitrogen), respectively. The cells were washed, fixed with 4% paraformaldehyde for 10 min, washed again, and mounted on coverslips. The samples were examined using a Nikon A1R laser scanning confocal microscope with a 100x oil objective. Images were analyzed using Nikon’s Elements software, version 4.1.

### Liposome preparation

The lipids used in this work, 1,2-dimyristoyl-*sn*-glycero-3-phosphocholine (DMPC) and 1,2-dioleoyl-*sn*-glycero-3-phosphoethanolamine-N-methyl (NmDOPE), were purchased from Avanti Polar Lipids (Alabaster, AL), and the sterols, cholesterol and cholesterol sulfate (CS), were purchased from Sigma-Aldrich (St. Louis, MO). Stock solutions of lipids dissolved in chloroform were added to a glass vial in the required amounts. The chloroform was evaporated under nitrogen, and any residual chloroform was removed under vacuum, resulting in a thin lipid film on the glass surface. The lipid film was hydrated with buffer and the resulting liposomes were extruded through a 200-nm polycarbonate membrane. For the tryptophan quenching experiments, the lipids were hydrated in a buffer containing 10 mM HEPES, 1 M NaCl, 10 mM CaCl_2_, and 1 mM Na_2_S_2_O_3_, at a pH of 7.4. Liposomes used in the mass spectrometry (MS) experiment were hydrated in 50 mM NH_4_HCO_3_ at a pH of 8.0.

### Tryptophan quenching

LtxA in solution or incubated with liposomes at a molar lipid-to-protein ratio (L:P) of 1000:1 was analyzed by fluorescence spectroscopy. A series of emission scans (300–400 nm) were collected, with an excitation wavelength of 280 nm and slit widths of 4 nm. The fluorescence intensity of the tryptophan at a wavelength of 350 nm was recorded. This intensity represents the intensity of all five Trp residues in LtxA. A baseline reading (F_o_) was obtained, and then a small volume (4 uL) of KI buffer (10 mM HEPES, 1 M KI, 10 mM CaCl_2_, 1 mM Na_2_S_2_O_3_) was added, and the sample was incubated for one minute before the fluorescence intensity (F) was recorded again. This process was repeated until the final KI concentration in the cuvette reached 200 mM.

The change in tryptophan intensity with addition of KI was determined by plotting F_o_/F as a function of KI concentration, where F_o_ is the intensity of tryptophan before the addition of KI and F is the tryptophan intensity after each addition of KI. The data were plotted on a Stern-Volmer plot following the equation:

$$\frac{F_{o}}{F}=1+K_{SV}[Q]$$

where K_SV_ is the Stern-Volmer constant, and [Q] is the concentration of quencher (I^-^). The slope of each line represents K_SV_, which is a measure of quenching efficiency and thus, the solvent accessibility of the Trp residues.

### Trypsin digest

First, LtxA was incubated with buffer or DMPC liposomes at a molar L:P of 1000:1 for 30 mins. Unbound toxin was separated from the liposomes by centrifugal filtration using a Pall 300,000 molecular weight cut-off (MWCO) filter. The proteoliposome solution was added to a Vivacon 500 30,000 MWCO filter and centrifuged at 13,500 x g for 10 min. Additional buffer was added, and the centrifugation step was repeated twice. Next, the digest was initiated by adding 50 uL of 0.04 ug/uL trypsin (enzyme-to-protein ratio of 1:50) to the filter unit, which was then shaken for 1 min. The filter units were incubated in a water bath at 37 °C for 1 hr. After the 1-hr digestion, the filter units were centrifuged at 14,000 x g for 10 min to separate the digested peptides from the proteoliposome. Additional buffer was added, and the centrifugation step was repeated twice. Ten uL of 10% formic acid was added to the filtrate to stop the trypsin digestion. The filtered material was analyzed by mass spectrometry to identify the peptides derived from the domains of LtxA that reside outside of the membrane.

The unfiltered material (liposomes and undigested toxin) was removed from the centrifugal filter and run on a Novex NuPAGE 12% Bis-Tris gel with NuPAGE MES SDS Running Buffer until completion. A BenchMark Pre-Stained Protein Ladder was used to determine the molecular weight of the peptides. The major bands were excised, digested with trypsin, and analyzed by mass spectrometry to identify the peptides derived from the domains of LtxA residing within the liposome.

### Mass spectrometry

LC-MS/MS analyses were performed at the Wistar Proteomics and Metabolomics Facility (Philadelphia). Trypsin digestions were loaded into a UPLC Symmetry trap column (180 μm i.d. × 2 cm packed with 5 μm C18 resin; Waters), and separations were performed on a 15-cm PicoFrit column (75 μm inner diameter, New Objective) packed with Magic 5 μm C18 reversed-phase resin (Michrom Bioresources) using a nanoflow high-pressure LC system (Eksigent Technologies), which was coupled online to a hybrid LTQ-Orbitrap XL mass spectrometer (Thermo Fisher Scientific) via a nanoelectrospray ion source. Chromatography was performed with Solvent A (Milli-Q water with 0.1% formic acid) and Solvent B (acetonitrile with 0.1% formic acid). Peptides were eluted at 200 nL/min for 3–28% B over 40 min, 28–50% B over 25.5 min, 50–80% B over 5 min, hold at 80% B for 4.5 min before returning to 3% B over 1 min. To minimize sample carryover, a short blank gradient was run between each sample. The LTQ-Orbitrap XL was operated in the data-dependent mode to automatically switch between full scan MS (m/z 300–2000) in the Orbitrap analyzer (with resolution of 60,000 at m/z 400) and the fragmentation of the six most intense ions by collision-induced dissociation in the ion trap mass analyzer.

MS/MS spectra were extracted and searched using the SEQUEST algorithm in BioWorks (version 3.3, Thermo Fisher Scientific) against a custom database containing the LtxA protein sequence, combined with a reverse database and a list of common contaminants. MS/MS spectra were searched using partial trypsin specificity with up to two missed cleavages, a 100 ppm precursor mass tolerance, 1 amu fragment ion mass tolerance, static modification of cys by carbamidomethylation (+57.0215), and variable modifications for methionine oxidation (+15.9949) and asparagine deamidation (+0.9840). Consensus protein lists were generated by DTASelect (version 2.0, Scripps Research Institute, La Jolla, CA).

### Dot blot

For dot blot analysis, the LtxA digestion was performed in a similar manner as in the MS experiment, but the resulting digested peptides were analyzed by immunoblotting rather than MS. First, LtxA was incubated with trypsin to digest the entire protein. Two uL of this peptide solution, representing peptides derived from the entire LtxA molecule, was spotted onto three separate nitrocellulose membranes (top dot). Next, LtxA was incubated with DMPC, the unbound protein was removed, and the proteoliposomes were incubated with trypsin to digest the domains of the toxin residing outside of the membrane. The resulting peptides were separated from the liposomes using a centrifugal filter, and two uL of this peptide solution was spotted onto the nitrocellulose membranes (middle dot). Finally, two uL of the remaining liposome solution, containing undigested domains of LtxA, representing the domains of the toxin that reside inside the membrane, was spotted onto the nitrocellulose membranes (bottom dot). The membranes were allowed to dry, blocked with Blotto [39], and then incubated with one of three monoclonal antibodies to LtxA, mAb28, mAb83, or mAb107 [14] at 4 °C overnight. These antibodies recognize epitopes located throughout the sequence of LtxA (mAb28: 698–709; mAb83: 746–757; mAb107: 926–937), as shown in Fig 1. The membranes were then incubated with goat anti-mouse-horseradish peroxidase (GAM-HRP) for one hour, and the chemiluminescence was analyzed on a Kodak Image Station. The pixel intensity of the dots was analyzed using ImageJ, with the average background pixel intensity subtracted.

### Statistical analysis

Data are presented as the mean ± standard deviation. Statistical analysis was performed using an unpaired two-tailed student’s t-test, where p values less than 0.05 were considered to be statistically significant.

## Results

### LtxA interacts with the host cell membrane

**Fig 2:**
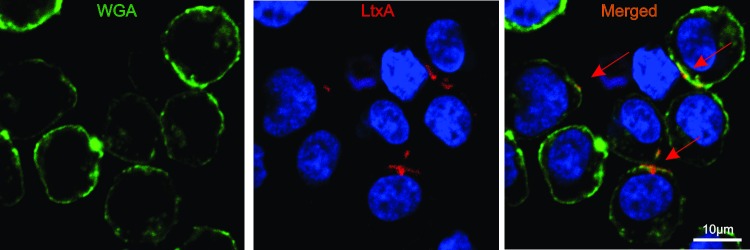
LtxA associates with the plasma membrane of Jn.9 cells. Confocal images of Jn.9 cells treated with 20 nM LtxA-DY650 (red) for 15 min show evidence of membrane localization by the toxin. The membranes were stained with WGA-AF488 (green) and the cell nuclei with Hoescht (blue). Arrows demonstrate areas of colocalization of LtxA with the membrane.

Jn.9 cells express cell-surface LFA-1 and are susceptible to LtxA [26]. Confocal images of Jn.9 cells in which the membranes were stained with WGA-AF488 (green) showed evidence of membrane localization of LtxA (LtxA-DY650, red) after a 15 min treatment (Fig 2). Five regions of interest were selected in areas of strong LtxA fluorescence, and the Manders’ coefficient, M1, corresponding to the fraction of pixels containing LtxA that also contain WGA, was calculated to be 0.397 ± 0.021.

### LtxA adopts an altered conformation upon membrane interaction

To investigate the membrane association of LtxA, a tryptophan quenching experiment was performed. In this assay, iodide ions quench the fluorescence of specific tryptophan residues that are exposed to aqueous solution. Tryptophan residues that are sequestered from solution (because of protein folding or because of the protein’s association with a membrane) remain unquenched because they are inaccessible to the aqueous iodide ions. As shown in Fig 1, LtxA contains five Trp residues, spaced throughout the sequence, W^115^, W^430^, W^478^, W^577^, and W^901^. This experiment thus allowed us to predict changes in LtxA folding as the toxin associates with the membrane environment.

Soluble LtxA and membrane-associated LtxA were exposed to increasing concentrations of KI, and a Stern-Volmer plot was constructed from the fluorescence intensity of the Trp residues in LtxA as a function of KI concentration. The Stern-Volmer constant (K_SV_) was determined from the slope of the linear fit to each data set. K_SV_ represents the rate constant of quenching (k_q_) and the lifetime of unquenched Trp (τ_o_):

$$K_{SV}=k_{q}\tau_{o}$$

Thus, K_SV_ is related to the accessibility of the Trp residues to aqueous iodide ions.

**Fig 3:**
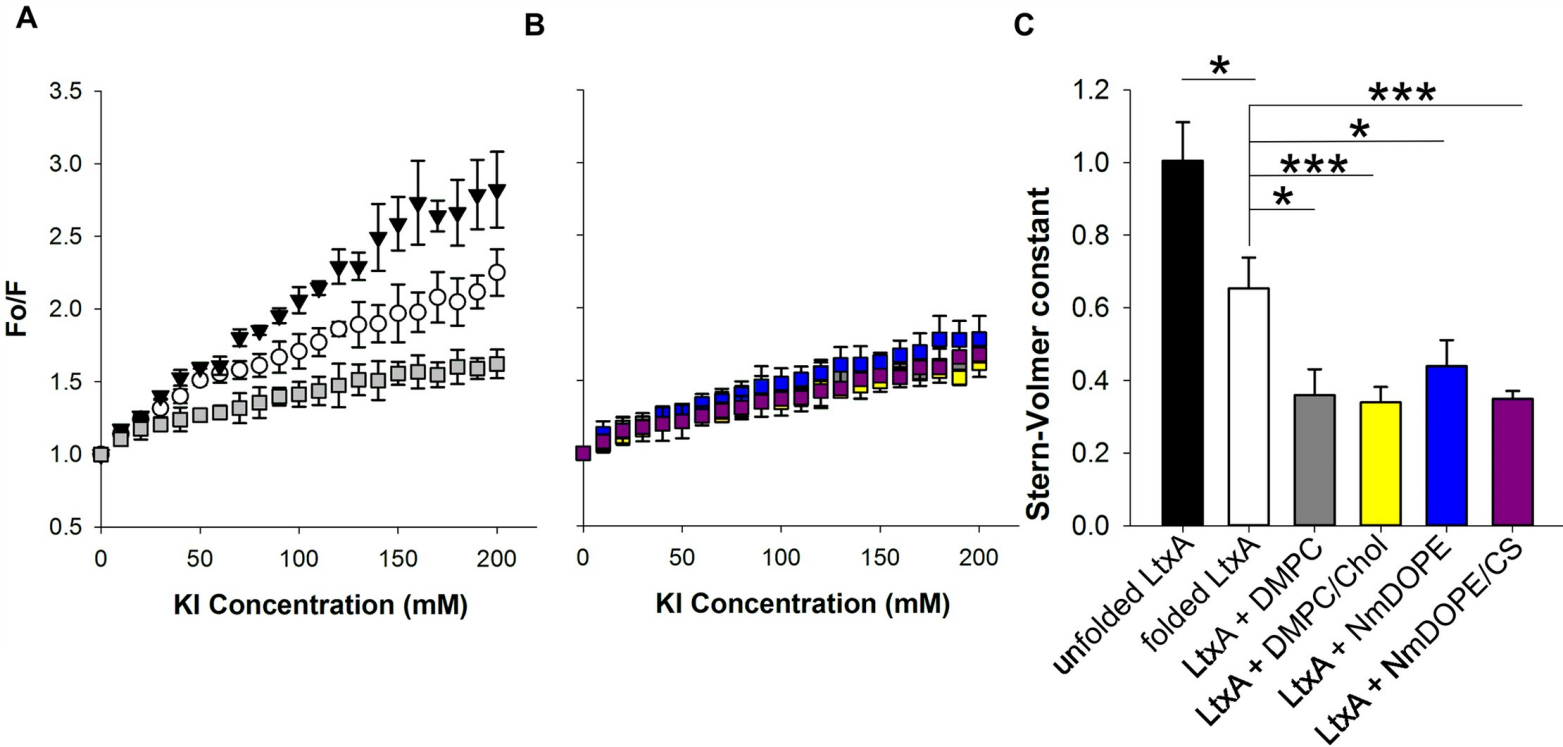
Tryptophan quenching of LtxA by KI. (A) Stern-Volmer plots, showing solvent accessibility of unfolded LtxA (black triangles), LtxA in solution (white circles), and LtxA incubated with DMPC (grey squares). The slopes of each line represent the Stern-Volmer constant (K_SV_), a measure of solvent accessibility of the five Trp residues within LtxA. (B) Stern-Volmer plots of LtxA in different membrane environments: DMPC (grey), DMPC + 40% Chol (yellow), NmDOPE (blue), and NmDOPE + 25% CS (purple). (C) Stern-Volmer constants (K_SV_) of the data shown in panels (A) and (B). In unfolded LtxA, K_SV_ is 1.0. In this unfolded state, 100% of the Trp residues are exposed to quencher; therefore, this value corresponds to 100% accessibility of the Trp residues. When LtxA is folded, K_SV_ decreases to 0.6, corresponding to 60% accessibility of the residues. When LtxA is incubated with membranes, regardless of the lipid composition, K_SV_ decreases to 0.4, indicating 40% accessibility of the residues. *, p < 0.05; ***, p < 0.005.

As a control, LtxA was first denatured using GuHCl. The Stern-Volmer plot of unfolded LtxA resulted in a K_SV_ that was calculated to be 1.00 mM^-1^ (Fig 3A). In this unfolded state, all five Trp residues are accessible to the quencher; we therefore considered this K_SV_ value to be equivalent to a conformation in which the Trp residues are 100% accessible to quencher. We compared all other K_SV_ values to this to obtain the fraction accessibility of the Trp residues. The Stern-Volmer plot of folded LtxA in solution was linear, with a slope of 0.65 mM^-1^, indicating that the Trp residues are approximately 60% accessible to the aqueous environment, or that three of the five resides are exposed to solution in this folded state. When LtxA was incubated with DMPC membranes, the slope decreased to 0.36 mM^-1^, indicating that two of the five Trp residues (approximately 40%) are exposed to solution, and three residues are inaccessible to the aqueous quencher. We interpret this change to suggest that these three residues interact with the liposome, consistent with a conformational change in LtxA as it moves from a water-soluble to membrane-soluble state.

To determine if the membrane-associated conformation depends on lipid composition, we repeated the quenching experiment using a variety of lipid compositions. These lipid compositions included 100% DMPC and 60% DMPC/40% Chol, a composition for which we have demonstrated LtxA has a large affinity [24], as well as 100% NmDOPE, which LtxA is able to disrupt, and 75% NmDOPE/25% CS, which LtxA is unable to disrupt [25]. As shown in Fig 3B, the Stern-Volmer plots of LtxA incubated with each of four different lipid compositions are quite similar, and K_SV_ values ranged from 0.34 to 0.44 (Fig 3C), with no statistically significant differences between the values, indicating that within these defined lipid compositions, two of the five Trp residues are exposed to solution when LtxA is in its membrane-bound state.

### LtxA is located partially embedded in the membrane

**Fig 4:**
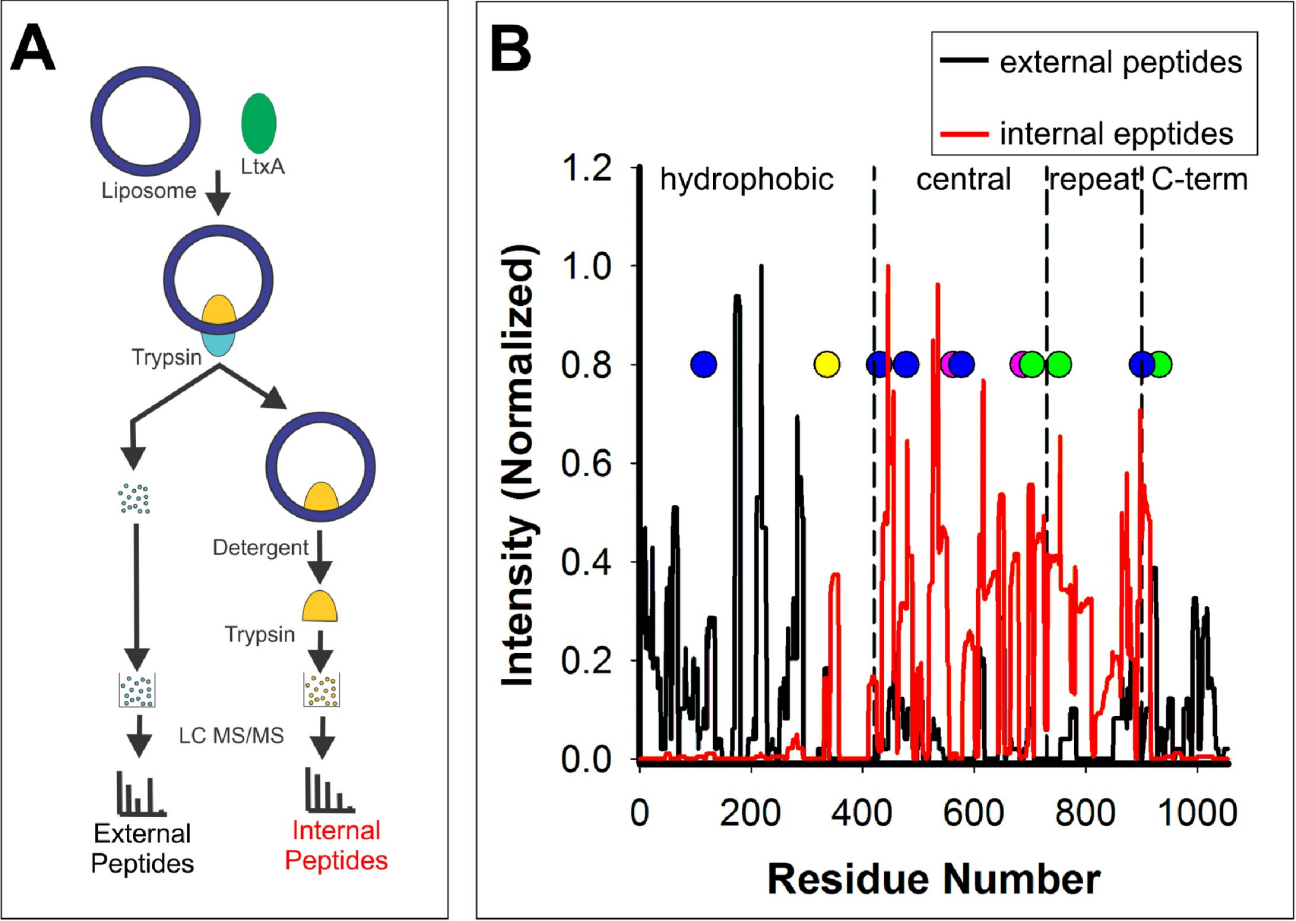
Mass spectrometry analysis of LtxA in solution and in DMPC membranes. Mass spectrometry was used to analyze the results of a trypsin digest experiment. (A) Schematic of the experimental design. LtxA was incubated with DMPC liposomes. The portion of the protein residing outside of the membrane was digested with trypsin; the resulting peptides were separated from the proteoliposome and analyzed by MS. Next, the remaining liposomes, which contain only the domains of LtxA residing inside the membrane were run on an SDS-PAGE gel (which disrupts the liposome); the major protein bands were excised, digested with trypsin and analyzed by MS. (B) Summary of the MS results. Together, the MS results indicate that the hydrophobic and C-terminal domains of LtxA reside outside of the membrane (black), while the central and repeat domains reside within the liposome (red). In each MS plot, the cholesterol-binding motif (yellow), acylation sites (pink), tryptophan residues (blue), and mAb epitopes (green) are marked.

The tryptophan quenching experiment suggested that LtxA may be partially embedded in the membrane. To identify which domains are protected by the membrane, a trypsin digest/mass spectrometry experiment was performed. A schematic of the experiment is shown in Fig 4A. Briefly, LtxA was incubated with liposomes before being exposed to trypsin to digest the domains of the toxin residing outside of the membrane. These digested peptides were separated and analyzed by MS. The remaining liposomes were collected, and the domains of LtxA still associated with the liposomes were separated by SDS-PAGE, digested by trypsin, and analyzed by MS. The timing of the digestion was optimized, and a one-hour incubation with trypsin was determined to be sufficient to distinguish between domains located within and external to the membrane.

Initially, free LtxA (not incubated with liposomes) was digested by trypsin and analyzed by MS to determine the effectiveness of this technique in detecting domains throughout the entire toxin structure. In this control experiment, peptides were detected throughout the length of the protein, with a coverage of 84% (S2 Fig), demonstrating that MS is able to detect a majority of the full-length protein.

To determine which portions of the toxin were solvent-exposed after liposome-association, LtxA was incubated with DMPC liposomes and exposed to trypsin to digest only the domains of LtxA located external to the membrane. MS analysis of the digested portion of the toxin is shown in S3 Fig and indicates that many of the resulting peptides were derived from the hydrophobic and C-terminal domains of LtxA, with fewer detected in the central and repeat domains.

Next, the domain(s) of LtxA that were inaccessible to trypsin digestion, likely membrane associated, were analyzed by MS. After trypsin digestion of the exposed domains of LtxA, SDS-PAGE analysis of the resulting LtxA-liposome detected four primary protein bands, with molecular weights of 70, 50, 25, and 20 kDa (Panel A in S4 Fig), which were excised and digested with trypsin. Most of the peptides from the 70 and 50 kDa bands were located in the central and repeat domains of LtxA, while the peptides from the 25 and 20 kDa bands were located primarily in the central domain, as shown in Panel B in S4 Fig. Few of the detected peptides from any of the bands were located in the hydrophobic or C-terminal domains.

Together, the MS results indicate that the hydrophobic and C-terminal domains of LtxA are accessible to trypsin, thus likely located external to the membrane, while the central and repeat domains are protected from the trypsin and thus likely interact with the membrane (Fig 4B). This suggests that in a membrane environment, LtxA adopts a conformation in which the N- and C-terminal ends of the molecule are located outside of the membrane while the interior portion of the protein is located within the liposome.

To further verify our results, we took advantage of three monoclonal antibodies which recognize epitopes that are located throughout the protein [14]. The epitope of antibody mAb28 resides between residues 698–709 (central domain), the epitope of antibody 83 resides between residues 746 and 757 (repeat domain), and the epitope of antibody 107 resides between residues 926–937 (C-terminal domain). Based on the results of the Trp quenching and MS experiments, the epitopes to mAb 28 and 83 are expected to be located within the membrane and thus protected from the trypsin digestion, while the epitope to mAb 107 would be exposed and subject to trypsinolysis.

**Fig 5:**
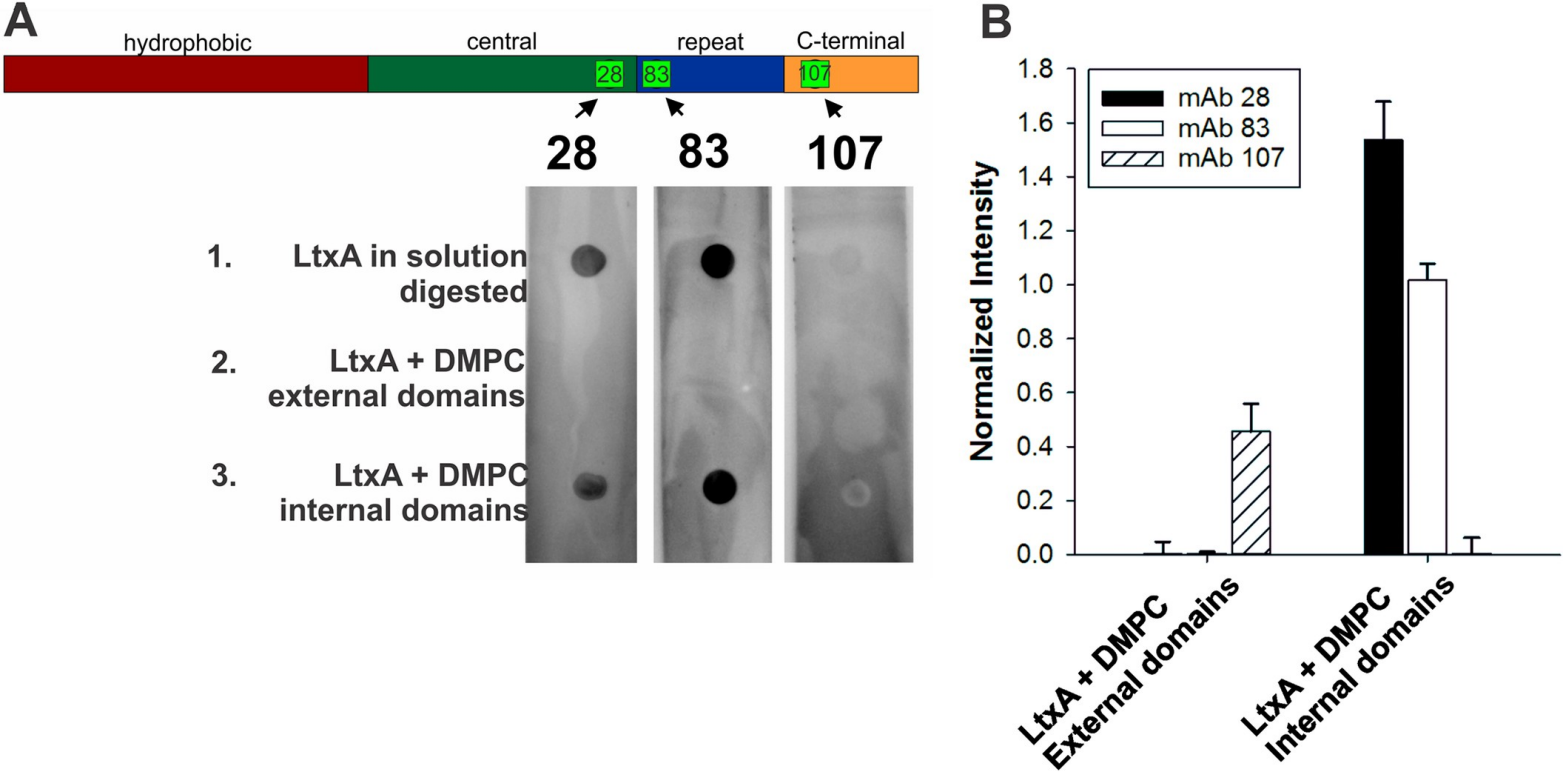
Dot blot analysis of exposed (external) and protected (internal) regions of LtxA in solution and in DMPC membranes. (A) Dot blot analysis of 1) trypsin-exposed LtxA, 2) digested domains of LtxA-liposomes, and 3) undigested domains of LtxA-liposomes. (1) Full-length, digested LtxA was used as a control (Dot #1). Antibodies 28 and 83 were able to detect their epitopes in full-length LtxA; however, the epitope for antibody 107 was digested in the typsin treatment and therefore, undetectable. (2) LtxA was incubated with DMPC liposomes for 30 mins and then digested with trypsin. The peptides resulting from this digestion, derived from domains of LtxA residing outside of the liposome, were separated and spotted on the nitrocellulose (Dot #2). None of the antibodies detected their epitopes, indicating that the epitopes for mAbs 28 and 83 reside inside the membrane. The epitope of mAb 107 is not detected, indicating either it is digested (thus, residing outside of the liposome) or is protected inside the liposome. (3) Finally, the liposomes, containing the domains of LtxA residing inside the liposomes were spotted on the nitrocellulose (Dot #3). Antibodies 28 and 83 detected their epitopes, confirming that these two epitopes are located in a domain of LtxA that is located inside the liposome. The epitope to mAb 107 was not detected, indicating that this epitope is not located within the membrane, and must therefore be located outside of the membrane. (B) Quantification of dot blot intensities, determined using ImageJ. Each bar represents detection of LtxA by one of three monoclonal antibodies, mAb 28 (black), mAb 83 (white), mAb 107 (hashed). Each intensity is normalized by the intensity of the digested LtxA in solution dot detected by the respective antibodies.

The trypsin digest experiment was performed as described above for MS analysis. The resulting peptide solutions were dotted onto a nitrocellulose membrane, which was then incubated with one of the three monoclonal antibodies, as shown in Fig 5. Dot #1 was composed of digested LtxA in solution (without liposomes). Dot #2 was composed of the peptides collected after trypsinolysis, representing the domains of the toxin exposed to solution. Dot #3 was composed of the domains of LtxA protected from digestion, representing the domains of the toxin that interact with the membrane. Undigested LtxA in solution and buffer controls were used as well (data not shown) to show maximal binding of the three antibodies and absence of antibody binding, respectively.

Initially, we looked at the ability of each peptide to detect trypsin-digested LtxA. As shown in Fig 5, although all three antibodies were able to detect undigested LtxA in solution, only antibodies mAb 28 and mAb 83 were able to detect digested LtxA in solution; mAb 107 could not (Dot #1). This result demonstrates that while the epitopes for mAb 28 and mAb 83 are not affected by the trypsin digest, the epitope of mAb 107 is digested by the trypsin because of its numerous Lys and Arg residues (S1 Table), thus preventing recognition by the antibody of this domain after exposure to trypsin.

After incubation with DMPC liposomes and digestion of all exposed portions of LtxA by trypsin, mAb 28 and mAb 83 recognized only the protected domains of LtxA (Dot #3) but not the exposed domains of LtxA (Dot #2), suggesting that the epitopes to these two antibodies are located in the membrane-interacting domains of LtxA (central and repeat domains). Antibody 107 did not detect any LtxA in this experiment. Because the epitope of mAb 107 is susceptible to trypsin digestion (Dot #1), we interpret this lack of antibody recognition to indicate that this epitope is digested by trypsin, and thus located external to the membrane (C-terminal domain). Together, the dot blot results corroborate the MS results, indicating that the C-terminal domain in which the epitope to antibody 107 is located resides external to the membrane, while the central and repeat domains, containing the epitopes to antibodies 28 and 83, are located within the membrane, where they are protected from trypsin digestion.

## Discussion

The RTX toxins are both water- and membrane-soluble, and therefore must undergo a conformational change to sequester hydrophilic residues when in the membrane-bound state [7, 40]. Interestingly, when we previously measured conformational changes in LtxA upon movement from the aqueous to membrane state, we found very small changes in secondary structure [41], suggesting that the structural changes that occur during this process are localized to specific, short domains of LtxA. In addition to these small-scale, localized changes in the secondary structure, our current data indicate that the toxin’s tertiary structure may change dramatically to accommodate the change in polarity upon binding to the membrane. These changes appear to be independent of lipid-composition and occur in the absence of LFA-1.

**Fig 6:**
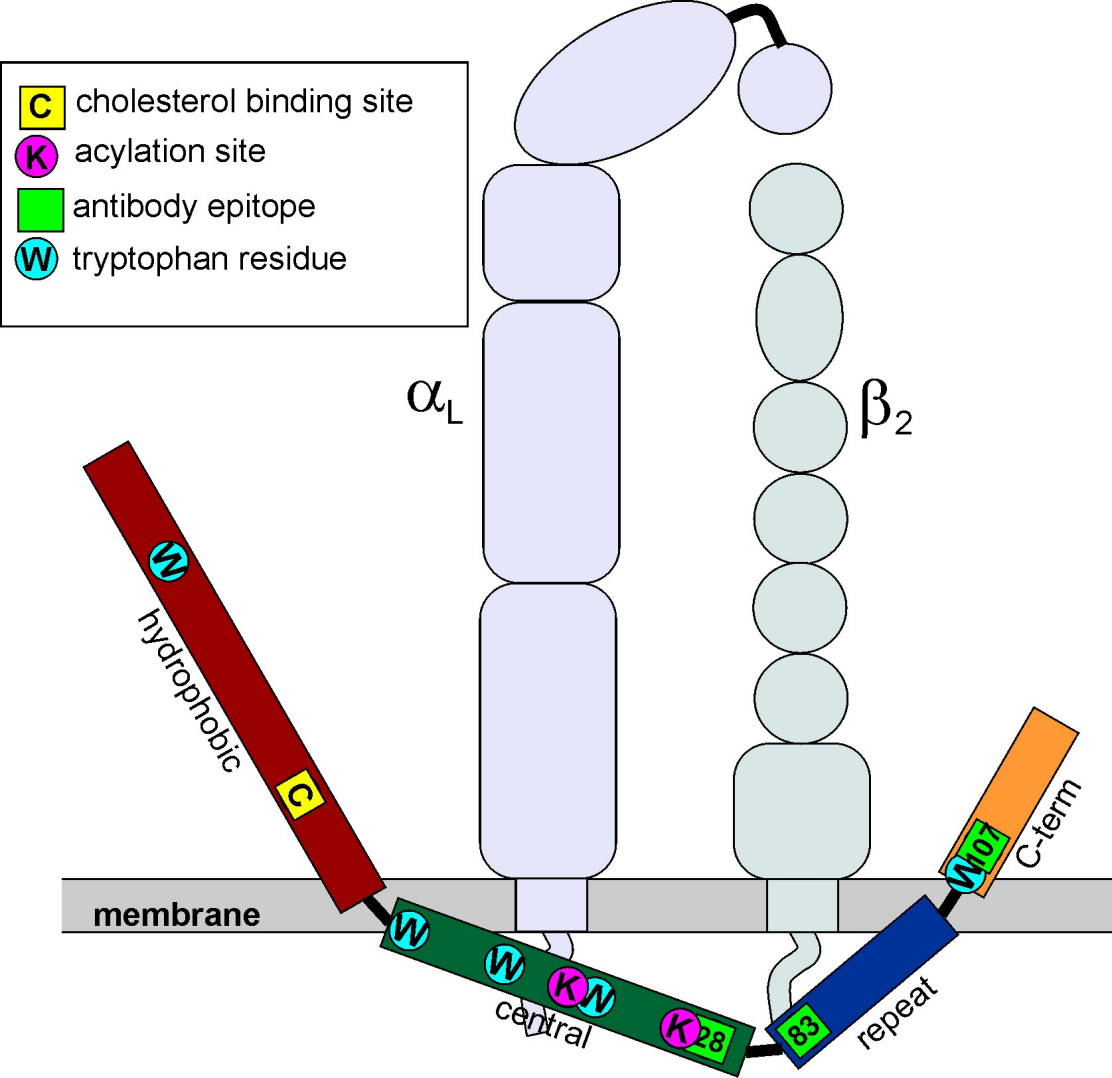
Location of LtxA in membrane. Together, the results indicate that LtxA adopts a U-shaped conformation, with the hydrophobic and C-terminal domains residing outside of the membrane. The MS results indicate that the hydrophobic and C-terminal domains reside external to the membrane, while the central and repeat domains reside inside the membrane. This finding was confirmed by dot blot analysis using three monoclonal antibodies, which demonstrated that the epitopes for mAb 28 and 83 reside inside the membrane and the epitope for mAb 107 resides outside of the membrane. The tryptophan quenching experiment indicated that two of the five tryptophan residues reside external to the membrane; based on the MS results, these residues are W^115^ and W^901^, located in the hydrophobic and C-terminal domains, respectively. W^430^, W^478^, and W^577^, located in the central domain are located inside the liposome. The figure also shows the subunits of LFA-1 (αL, purple; β2, green) to demonstrate which domains of LtxA may interact with the extracellular and intracellular domains of LFA-1.

In these studies, we have used three assays of aqueous accessibility to identify the domains of LtxA that are exposed to solvent and thus reside outside of the plasma membrane. The tryptophan quenching experiments indicated that in the membrane-bound conformation, two of the five Trp residues of LtxA are exposed to the aqueous environment. This finding is consistent with the trypsin digest/MS experiment, which demonstrated that the hydrophobic domain and C-terminal domain, each of which contains one Trp residue, are exposed to the aqueous environment when the toxin is incubated with DMPC liposomes. Additionally, the epitopes to two monoclonal anti-LtxA antibodies, located in the central and repeat domains, were found to be protected from trypsinolysis, while the epitope to a third monoclonal antibody located in the C-terminal domain was exposed to aqueous trypsin. Collectively, these three experiments suggest that LtxA associates with the membrane in a conformation in which the hydrophobic and C-terminal domains reside outside of the membrane and the central and repeat domains inside (Fig 6). Similar results were obtained in liposomes with four distinct lipid compositions, indicating that the conformation may be insensitive to the presence of cholesterol or nonlamellar phase-forming lipids.

The specific transmembrane (TM) domains of the membrane-embedded conformation of LtxA most likely reside between the hydrophobic and central domains and between the repeat and C-terminal domains. One clue to the identity of the N-terminal transmembrane domain comes from the MS analysis of the full-length, soluble LtxA. Transmembrane domains often do not contain sites for protease digestion [42] and are helical in nature [43]. In our MS analysis, we identified a long undigested domain between residues 368 and 412, which contains hydrophobic residues, including a number of alanine residues, which promote helix formation [44, 45]. Because this domain is located between the predicted external hydrophobic domain and the predicted internal central domain, it is quite likely that one of the two TM domains is found within this region.

One common structural feature of the RTX toxins is an N-terminal domain that contains a large number of hydrophobic residues; this domain has been hypothesized to play a role in the binding of the toxins to the lipid bilayer [2]. Here, we have found that the hydrophobic domain of LtxA, however, resides external to the membrane and is therefore likely not involved in the reported membrane interactions of the toxin. This finding agrees with a previous report that the binding of α-HlyA to lipid bilayers is not regulated by the hydrophobic domain [46]. Previously, we found that a cholesterol recognition amino acid consensus (CRAC) motif is present within the hydrophobic domain and is essential for LtxA binding to cholesterol on the host cell plasma membrane and toxicity [24]. In the present work, this CRAC site was detected in both the intra- and extra-membrane peptides by MS, indicating that this site may be located very close to the membrane interface, consistent with a mechanism suggested by recent work in our lab [47, 48], demonstrating that LtxA recognizes cholesterol near the membrane interface.

In addition to cholesterol, LtxA must also recognize an integrin receptor, LFA-1, on the target cell membrane [26]. We have found that LtxA is able to interact with cell membranes that do not express LFA-1, but is unable to enter the cellular cytosol in the absence of this integrin receptor. The domains of LFA-1 that LtxA has been reported to interact with include the β-propeller of the α_L_ subunit [32, 33] and the I-EGF domains of the β_2_ subunit [34] as well as the cytosolic domains of both the α_L_ and β_2_ subunits [35]. The membrane-bound conformation suggested by our proteolysis studies suggests that LtxA may be able to simultaneously interact with cholesterol as well as the extra- and intracellular domains of LFA-1. We hypothesize that the toxin first inserts into the membrane to adopt the conformation described here; this structure facilitates binding to LFA-1. Previous work in our lab identified three monoclonal antibodies (mAb 28, mAb 83, and mAb 107) that inhibit LtxA-mediated cytotoxicity in HL-60 cells [14]. The epitopes recognized by mAb 83 (residues 746–757) and 107 (residues 926–937) were predicted to be involved in the recognition of LFA-1 by LtxA. We have shown here that the epitope recognized by mAb 83 is protected from trypsin digestion by the liposome, suggesting that it may interact with the cytosolic domains of LFA-1, while the epitope recognized by mAb 107 is digested by trypsin, suggesting it is located external to the membrane and may therefore interact with the extracellular domains of LFA-1, as shown in Fig 6.

In the predicted conformation, the two acylation sites of LtxA are located in a domain of the toxin that is located within the liposome. Consensus on the role of acylation in RTX toxins has not been reached, but it appears to play a role in membrane interactions, including cell association and lysis, more so than in toxin-receptor interactions [49–53]. In addition, the third monoclonal antibody epitope, recognized by mAb 28 (residues 698–709), which was shown to be involved in membrane, but not LFA-1, interactions [14], is located in this same region, in the putative membrane-interacting domain. While the specific role of these hydrocarbon chains on LtxA activity has not yet been identified, our results suggest that the acyl chains serve to initially anchor the protein to the membrane to initiate the membrane destabilization and conformational change observed here.

## Conclusions

We have presented evidence of a membrane-inserted conformation of LtxA that is consistent with previous reports of LtxA and RTX membrane interactions. Based on the structural and sequence similarities in this family of proteins, we predict that many of the RTX toxins may adopt a similar conformation in the membrane environment. Because this conformation is independent of lipid composition, we hypothesize that the interaction of the RTX toxins with their specific receptors determines the cell type specificity of the interaction, but that the membrane interaction is mostly consistent across the family.

## Supporting information

S1 Fig LtxA purification. LtxA was purified by ion-exchange chromatography, and the purity was analyzed by SDS-PAGE.
(TIF)

S2 Fig MS detection of full-length LtxA in solution. LtxA in solution was digested with trypsin, and the resulting peptides were analyzed by MS. Peptides across the entire sequence of LtxA were detected, with a coverage of 84%. In the plot, the cholesterol-binding motif (yellow), acylation sites (pink), tryptophan residues (blue), and mAb epitopes (green) are marked.
(TIF)

S3 Fig MS detection of peptides residing outside the DMPC liposome. LtxA was incubated with DMPC liposomes and then exposed to trypsin, resulting in digestion of only the domains of LtxA residing outside of the liposome. These peptides were separated and analyzed by MS. Most of the external peptides detected by MS were located in the hydrophobic and C-terminal domains. In the plot, the cholesterol-binding motif (yellow), acylation sites (pink), tryptophan residues (blue), and mAb epitopes (green) are marked.
(TIF)

S4 Fig MS detection of peptides residing within the DMPC liposome. After trypsin-digestion of the external peptides of LtxA, the remaining proteoliposomes containing only the domains of LtxA protected from digestion by the liposome were run on an SDS-PAGE gel (A). Four major bands at 1) 70 kDa, 2) 50 kDa, 3) 25 kDa, and 4) 20 kDa were excised and digested by trypsin. (B) The peptides from the four major bands in the SDS-PAGE gel were analyzed by MS. In the 70- and 50-kDa bands, the detected peptides resided in the central and repeat domains, and in the 25- and 20-kDa bands, the detected peptides resided in the repeat domain. In each plot, the cholesterol-binding motif (yellow), acylation sites (pink), tryptophan residues (blue), and mAb epitopes (green) are marked.
(TIF)

S1 Table mAb epitopes and trypsin digest sites. Trypsin digest sites are in bold and underlined.
(DOCX)
