# Supplementary figures and images for: Membrane localization of the Repeats-in-Toxin (RTX) Leukotoxin (LtxA) produced by Aggregatibacter actinomycetemcomitans
Source: PLoS One. 2018 Oct 18;13(10):e0205871. doi: 10.1371/journal.pone.0205871 (PMC6193665; doi:10.1371/journal.pone.0205871)

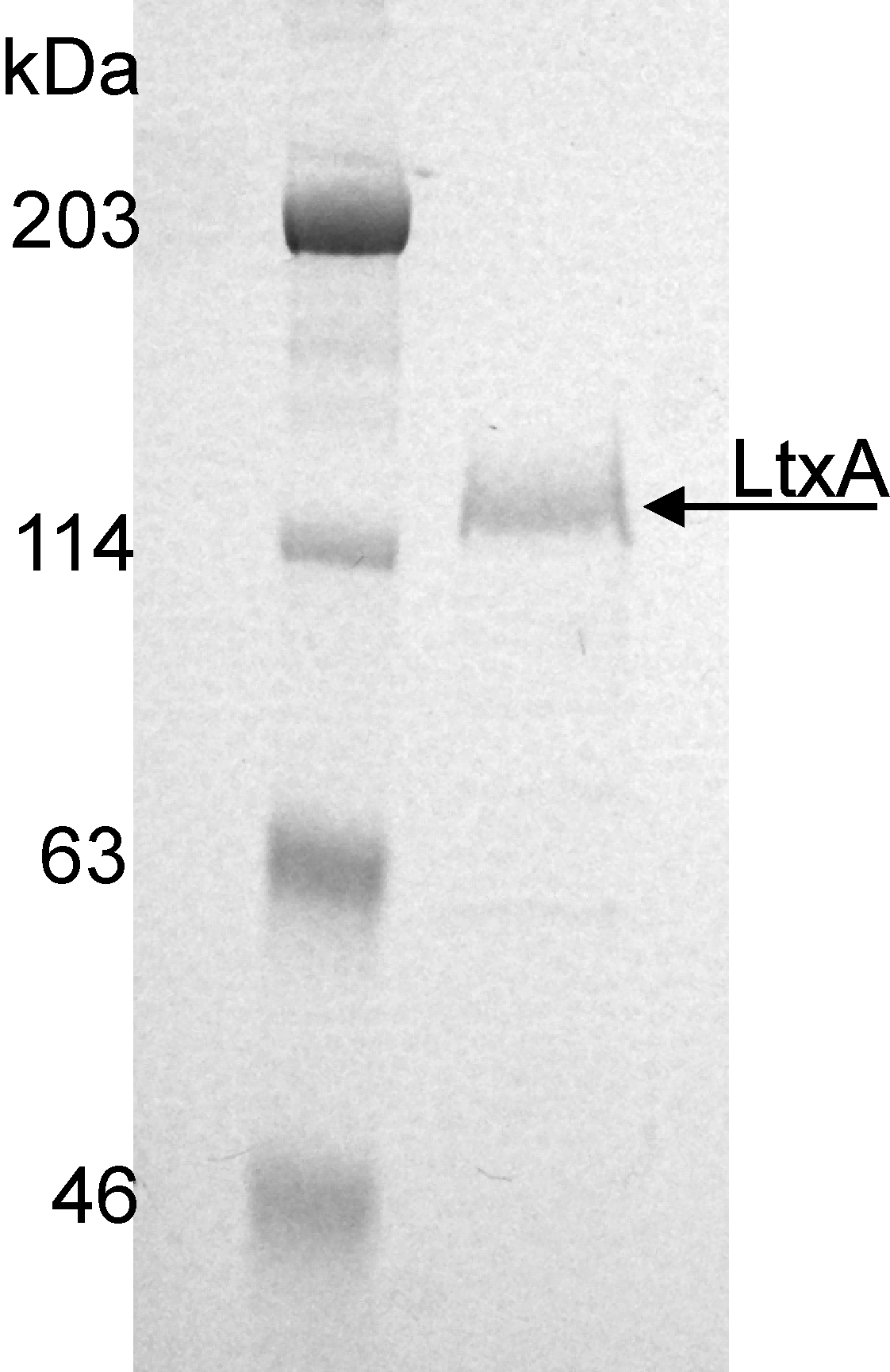

Supplement: S1 Fig — LtxA was purified by ion-exchange chromatography, and the purity was analyzed by SDS-PAGE. (TIF) [file pone.0205871.s001.tif]

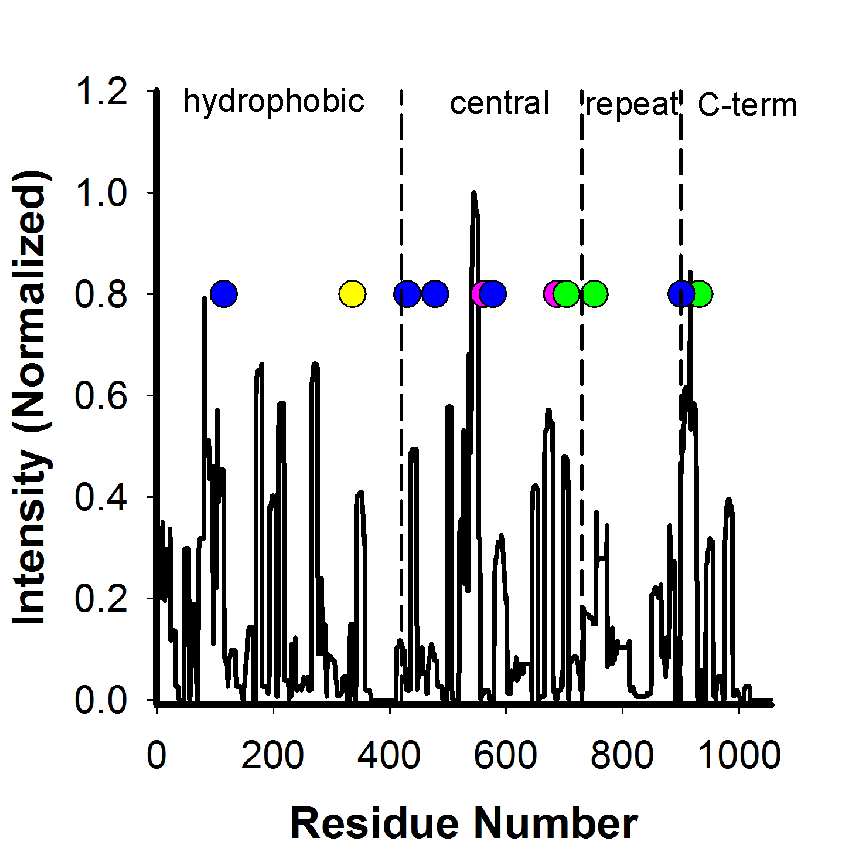

Supplement: S2 Fig — LtxA in solution was digested with trypsin, and the resulting peptides were analyzed by MS. Peptides across the entire sequence of LtxA were detected, with a coverage of 84%. In the plot, the cholesterol-binding motif (yellow), acylation sites (pink), tryptophan residues (blue), and mAb epitopes (green) are marked. (TIF) [file pone.0205871.s002.tif]

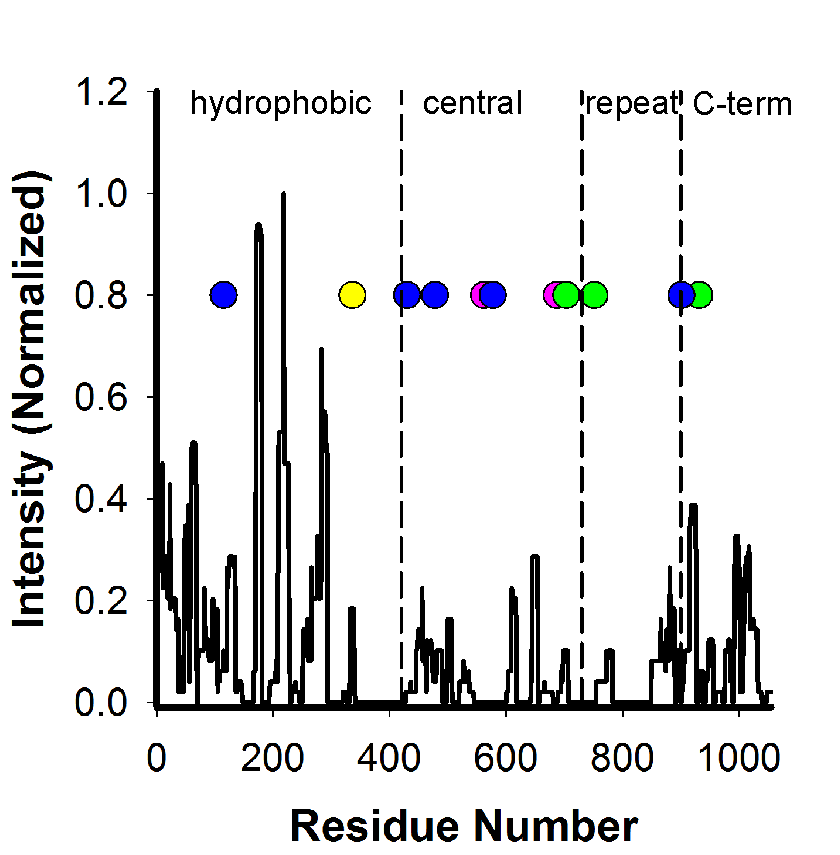

Supplement: S3 Fig — LtxA was incubated with DMPC liposomes and then exposed to trypsin, resulting in digestion of only the domains of LtxA residing outside of the liposome. These peptides were separated and analyzed by MS. Most of the external peptides detected by MS were located in the hydrophobic and C-terminal domains. In the plot, the cholesterol-binding motif (yellow), acylation sites (pink), tryptophan residues (blue), and mAb epitopes (green) are marked. (TIF) [file pone.0205871.s003.tif]

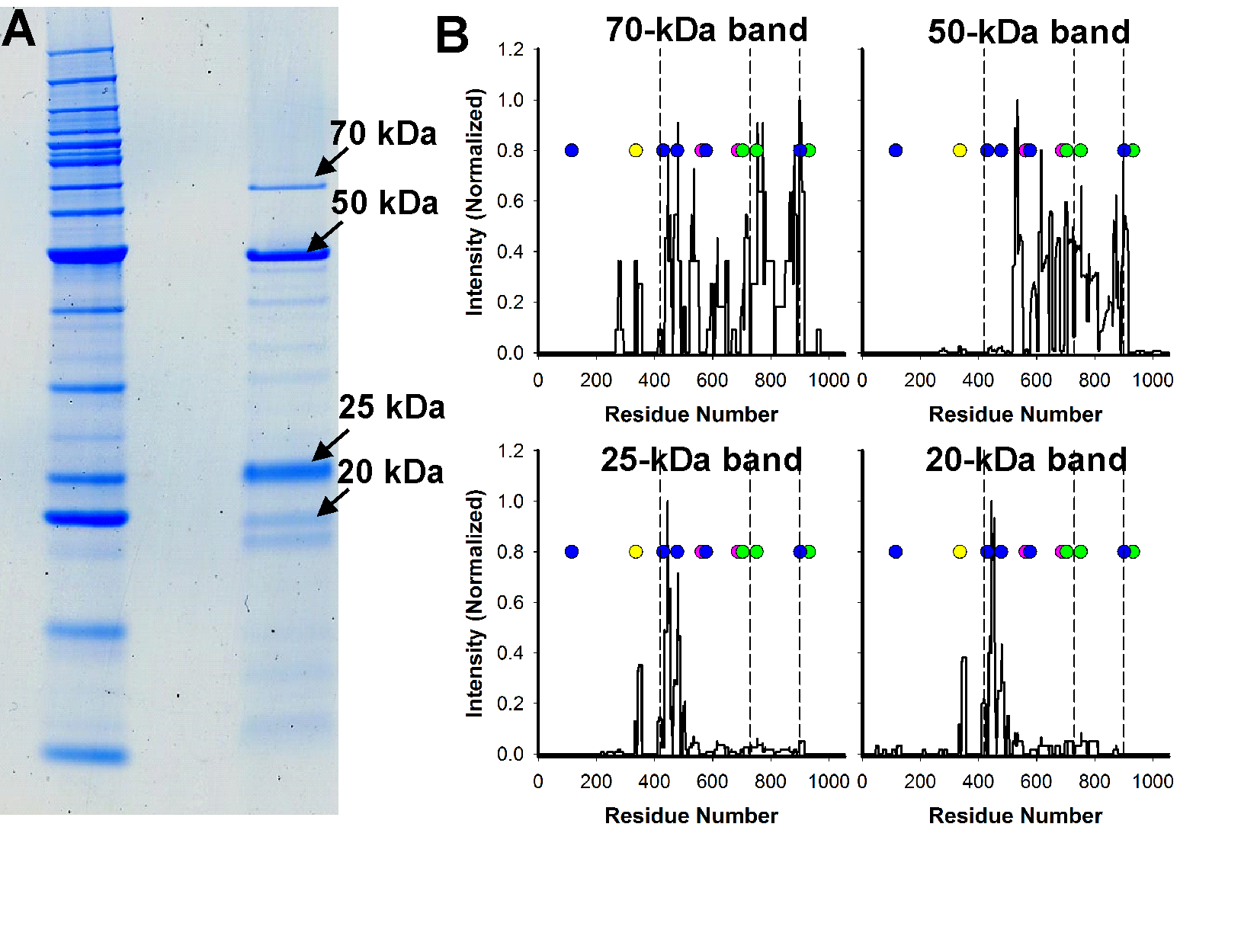

Supplement: S4 Fig — After trypsin-digestion of the external peptides of LtxA, the remaining proteoliposomes containing only the domains of LtxA protected from digestion by the liposome were run on an SDS-PAGE gel (A). Four major bands at 1) 70 kDa, 2) 50 kDa, 3) 25 kDa, and 4) 20 kDa were excised and digested by trypsin. (B) The peptides from the four major bands in the SDS-PAGE gel were analyzed by MS. In the 70- and 50-kDa bands, the detected peptides resided in the central and repeat domains, and in the 25- and 20-kDa bands, the detected peptides resided in the repeat domain. In each plot, the cholesterol-binding motif (yellow), acylation sites (pink), tryptophan residues (blue), and mAb epitopes (green) are marked. (TIF) [file pone.0205871.s004.tif]
