# Supplementary material for: Membrane localization of the Repeats-in-Toxin (RTX) Leukotoxin (LtxA) produced by Aggregatibacter actinomycetemcomitans
Source: PLoS One. 2018 Oct 18;13(10):e0205871. doi: 10.1371/journal.pone.0205871 (PMC6193665; doi:10.1371/journal.pone.0205871)
Supplement: S1 Table — Trypsin digest sites are in bold and underlined. (DOCX) [file pone.0205871.s005.docx]

**S1 Table.** mAb epitopes and trypsin digest sites. Trypsin digest sites are in bold and underlined.

| **mAb** | **Residue Numbers** | **Residues** |
| --- | --- | --- |
| mAb 28 | 698-709 | **K**LDYYYTN**K**GF**K** |
| mAb 83 | 746-757 | LIYGYDGDD**R**LY |
| mAb 107 | 926-937 | D**R**A**R**L**KR**QFELQ |
